# TLR7/8 agonist 3M-052 formulated in micelles induces local type I IFN response and antiviral immunity in zebrafish larvae

**DOI:** 10.3389/fimmu.2026.1834678

**Published:** 2026-06-16

**Authors:** Dean Porter, Hannah Wiggett, Axel Benchetrit, Pierre Boudinot, Bernard Verrier, Didier Gigmes, Thomas Trimaille, Jean-Pierre Levraud

**Affiliations:** 1 Université Paris-Saclay, Institut National pour l'Agriculture, l'Alimentation et l'Environnement (INRAE), Université Versailles Saint-Quentin (UVSQ), Virologie et Immunologie Moléculaire (VIM), Jouy-en-Josas, France; 2 Université Paris‐Saclay, Centre National de la Recherche Scientifique (CNRS) UMR9197, Institut Pasteur, Université Paris‐Cité, Institut des Neurosciences Paris‐Saclay, Saclay, France; 3 Université Paris-Saclay, Centre National de la Recherche Scientifique (CNRS) UAR 2010, TEFOR Paris-Saclay, Saclay, France; 4 Adjuvatis, Lyon-BioPole, Lyon, France; 5 Aix Marseille Univ, Centre National de la Recherche Scientifique (CNRS), Institut de Chimie Radicalaire, Marseille, France; 6 Université Claude Bernard Lyon 1, INSA Lyon, Université Jean Monnet, Centre National de la Recherche Scientifique (CNRS) UMR 5223, Ingénierie des Matériaux Polymères, Villeurbanne, France

**Keywords:** 3M-052, innate immunity, macrophage, micelle, neutrophil, TLR7/8 agonist, zebrafish, type I interferon

## Abstract

**Introduction:**

Micelle-based delivery systems provide a promising platform for vaccine adjuvants by encapsulating active compounds and enabling targeted delivery, thereby limiting systemic side effects. Here, we investigated the immune response, biodistribution, and antiviral effects of micelles loaded with the TLR7/8 agonist, 3M-052, in zebrafish (*Danio rerio*) larvae.

**Methods:**

Fluorescently labelled micelles were injected to transgenic reporter zebrafish larvae, and localization of micelles as well as neutrophils, macrophages, or mxa-expressing, was monitored by live confocal microscopy. Host response was also assessed at the whole-body level by qRT-PCR. In addition, larvae were infected with a recombinant Sindbis Virus to monitor the localization of viral infection.

**Results:**

Micelles demonstrated progressive clearance from the injection site and promoted local recruitment of neutrophils and macrophages, indicating immune activation. Whole-larva analysis showed no significant upregulation of key immune genes following unloaded or 3M-052-loaded micelle injection. However, 3M-052-micelles triggered a transient and localized mxa response, confirming induction of a local type I interferon response. Furthermore, zebrafish larvae exhibited significantly reduced virus replication near the site of 3M-052-micelle injection.

**Discussion:**

These findings indicate that micelles effectively deliver 3M-052 and elicit a localized antiviral response without broad systemic immune activation.

## Introduction

Understanding how adjuvants shape immune responses remains a critical challenge in vaccine development. Conventional models rely on endpoint analyses, limiting the ability to capture dynamic immune activation as it occurs. Zebrafish (*Danio rerio*) provide a unique model for real-time innate immune monitoring due to their transparent larvae and the availability of fluorescent reporter lines (1), which enable visualization of immune activation with real-time spatial and temporal analysis. Despite their established use in developmental biology and toxicology, zebrafish remain underutilized in vaccine research, even as the need for novel adjuvants and vaccine platforms has increased in response to emerging infectious diseases (2, 3). Zebrafish’s genetic tractability and conservation of key immune pathways, including toll-like receptor (TLR) mediated signaling and type I interferon (IFN) responses, make them particularly well suited for studying adjuvant mechanisms (4). Zebrafish larvae do not have an adaptive response at this age so allow for the direct responses of the innate immune system to micelles and adjuvants to be monitored.

Micelles are self-assembling amphiphilic copolymer nanoparticles, which offer a promising strategy for targeted immune modulation (5). Unlike traditional adjuvants, which rely on systemic immune activation, micelles facilitate the precise delivery of immunostimulatory molecules or vaccines, enhancing bioavailability and cellular uptake (6, 7). Importantly, they are not passive carriers; micelles can themselves promote immune activation through enhanced uptake by antigen-presenting cells promoting dendritic cell activation and antigen presentation (8, 9), giving them intrinsic adjuvant-like properties. When loaded with immunostimulatory ligands, micelles function as a composite adjuvant system rather than a simple delivery platform, in which both the carrier and the cargo may contribute to immune activation. In this configuration, micelles and incorporated ligands can act synergistically to enhance the magnitude and spatial control of innate immune responses. Additionally, micelles provide a versatile platform for tracking biodistribution, as they can encapsulate cargo within their hydrophobic core while enabling fluorophore conjugation to either the hydrophilic surface or the core itself (10, 11). Encapsulation of adjuvants also reduces premature immune activation, ensuring a more localized and controlled response (12, 13). However, their potential for adjuvant applications in fish remains unexplored.

Among immunostimulatory molecules, TLR7/8 agonists, such as 3M-052, have emerged as potent activators of type I IFN-based antiviral immunity. The natural ligands of the endosomal pattern recognition receptors TLR7 and TLR8, are single-stranded RNA and its degradation products (14). Their activation initiates a MyD88-dependent signaling cascade, leading to type I interferon production and pro-inflammatory cytokine release (15). There are many artificial TLR7/8 ligands, including base derivatives, and bi- or tri-cyclic compounds. The first synthetic agonists were imidazoquinolines Imiquimod and resiquimod, that have been extensively characterized, leading to the development of a broad repertoire of TLR7/8-targeting agonists and antagonists. In mammals, these compounds have shown promise as vaccine adjuvants for both infectious disease and cancer immunotherapy (16, 17).

3M-052 (also known as Telratolimod) is a lipid modified imidazoquinoline with immunostimulatory, antiviral and antitumoral activities. It forms tissue deposit in tissues or tumors, with sustained slow release, inducing local TLR7/8 triggering activity without systemic cytokine response in mice (18, 19). 3M-052 has been shown to trigger robust immune stimulatory activity in mammalian preclinical vaccine studies across a range of contexts (20). In newborn rhesus macaques, a 13−valent pneumococcal conjugate vaccine (PCV13) formulated with 3M−052 (0.1 mg/kg) elicited PnPS−specific IgG responses that were up to 100 times greater than those generated by PCV13 alone (21). In the context of HIV-1 immunization, 3M-052 induced durable antibody responses against the HIV-1 envelope protein, that resulted in 36% of bone marrow resident plasma cells being HIV-1 specific and remained detectable for one year (15). A receptor-binding domain (RBD)-sortase A-conjugated ferritin nanoparticle vaccine against SARS-COV2 induces antibody responses which are augmented and demonstrate broader protection against SARS-COV2 variants when adjuvanted with 3M-052-Alum (22). Further studies, using 3M-052 as the adjuvant and recombinant hemagglutinin from H1N1A/Puerto Rico/8/34 as a vaccine, reported strong Th1 responses and high levels of neutralizing antibodies without systemic induction of proinflammatory cytokines such as TNFα (19). The potent Th1-stimulating properties of 3M-052 have also been highlighted in vaccine studies against HIV-1 (23) and leishmaniasis (24), emphasizing its adjuvant potential. In addition to its adjuvant properties, 3M-052 exhibits antitumor activity by reshaping the tumor microenvironment, likely via IFN-mediated mechanisms (18). Upon injection 3M-052 has been shown to increase recruitment of M1 macrophages and induce CCL2 to the site of injection (25, 26). Although systemic antitumor effects have been inconsistent across models, 3M-052 has been reported to reduce melanoma burden even when administered locally and even distally from the tumor site.

Given the demonstrated efficacy of type I interferon-inducing adjuvants in fish vaccines (27, 28), micelle-based delivery of 3M-052 represents a novel approach for enhancing immune responses in aquatic species. In this study, we evaluated micelles encapsulating the TLR7/8 agonist, 3M-052, to assess their immunostimulatory effects and biodistribution *in vivo* in a non-mammalian vertebrate model. By using fluorescently labeled micelles and immune cell reporter lines in zebrafish, we tracked micelle clearance and characterized local immune cell recruitment dynamics. These analyses allowed us to examine whether micelle-based delivery can trigger innate immune responses without inducing broad systemic activation. This approach provides a valuable framework for investigating targeted adjuvant strategies in aquatic species and highlights the utility of zebrafish as a model for real-time immunological assessment.

## Results

### Biodistribution of micelles within zebrafish larvae

**Figure 1:**
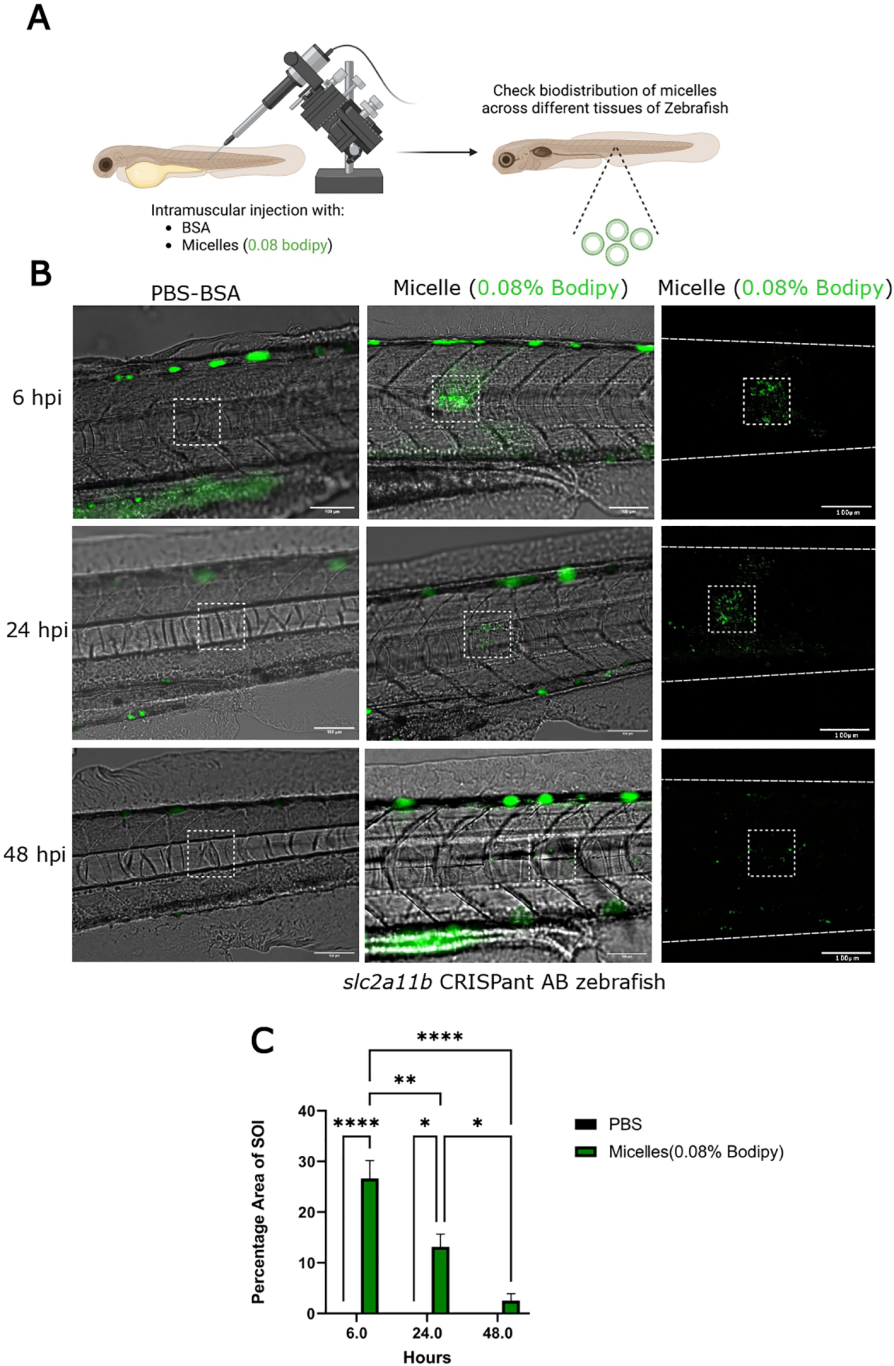
Biodistribution of micelles. **(A)** Schematic overview of the experimental design. **(B)** Biodistribution of micelles over 48 hours post-injection. *Slc2a11b* CRISPant AB zebrafish were injected with micelles (0.08% Bodipy, green), and their distribution was assessed at 6, 24, and 48 hours post-injection. Left and center columns: widefield imaging, merge of transmitted light and green fluorescence. Right column: confocal imaging, Z-stack of the muscle, not spanning the dorsal and ventral midlines with the main pigment cells, green fluorescence only. Representative images from 11 images larvae per group. **(C)** Bar plot showing mean with SEM quantifying the Bodipy signal within the site of injection (SOI) (White dashed box), expressed as a percentage of the total SOI area at each time point (*n* = 10). Statistical significance is indicated by * P < 0.05, ** P < 0.01, and **** P < 0.0001.

To assess the biodistribution of micelles, 3 days post fertilization (3dpf) zebrafish larvae were intramuscularly injected in the tail muscle with fluorescently labelled micelles and dispersion from the injection site was evaluated (**Figure 1A**). To reduce the green autofluorescence background due to xanthophore pigments, these experiments were carried out in *slc2a11b* CRISPants (**Supplementary Figure 1**). Intramuscular injection was chosen to replicate standard vaccine administration protocols in fish. Live widefield fluorescence imaging was used to track the biodistribution of micelles over a 48-hour period post-injection. To quantify micelles near the injection site, a 100µm-wide square centered on the point of injection was used. Fluorescence imaging of the tail showed a progressive reduction in the Bodipy signal at the injection site (**Figure 1B**). At 6 hours post injection (hpi), Bodipy fluorescence was significantly higher than in PBS-BSA injected controls (**Figure 1C**). At 24hpi, fluorescence within the injection site had decreased but remained significantly higher than in PBS-BSA injected controls. The right panel of **Figure 1B** shows a maximal projection of a Z-stack taken at the injection site via live confocal microscopy, clearly demonstrating partial dispersion of the Bodipy signal away from the injection site. At 48 hpi, no significant difference in the Bodipy signal was observed between micelle-injected and PBS-BSA-injected fish. Likewise, a clear reduction in the number of particles showing the Bodipy signal was evident at 48 hpi in the maximal projection of the Z-stack. This reduction in fluorescence and partial dispersal of the Bodipy signal may be attributed to immune cell activity, particularly macrophages and neutrophils, which are known to phagocytose and degrade foreign particles at the site of injection. Immune-mediated clearance could explain the progressive decrease in the Bodipy signal over time, as these cells likely contribute to both the degradation and dispersal of micelles.

### Recruitment of macrophages and neutrophils at micelles injection site

**Figure 2:**
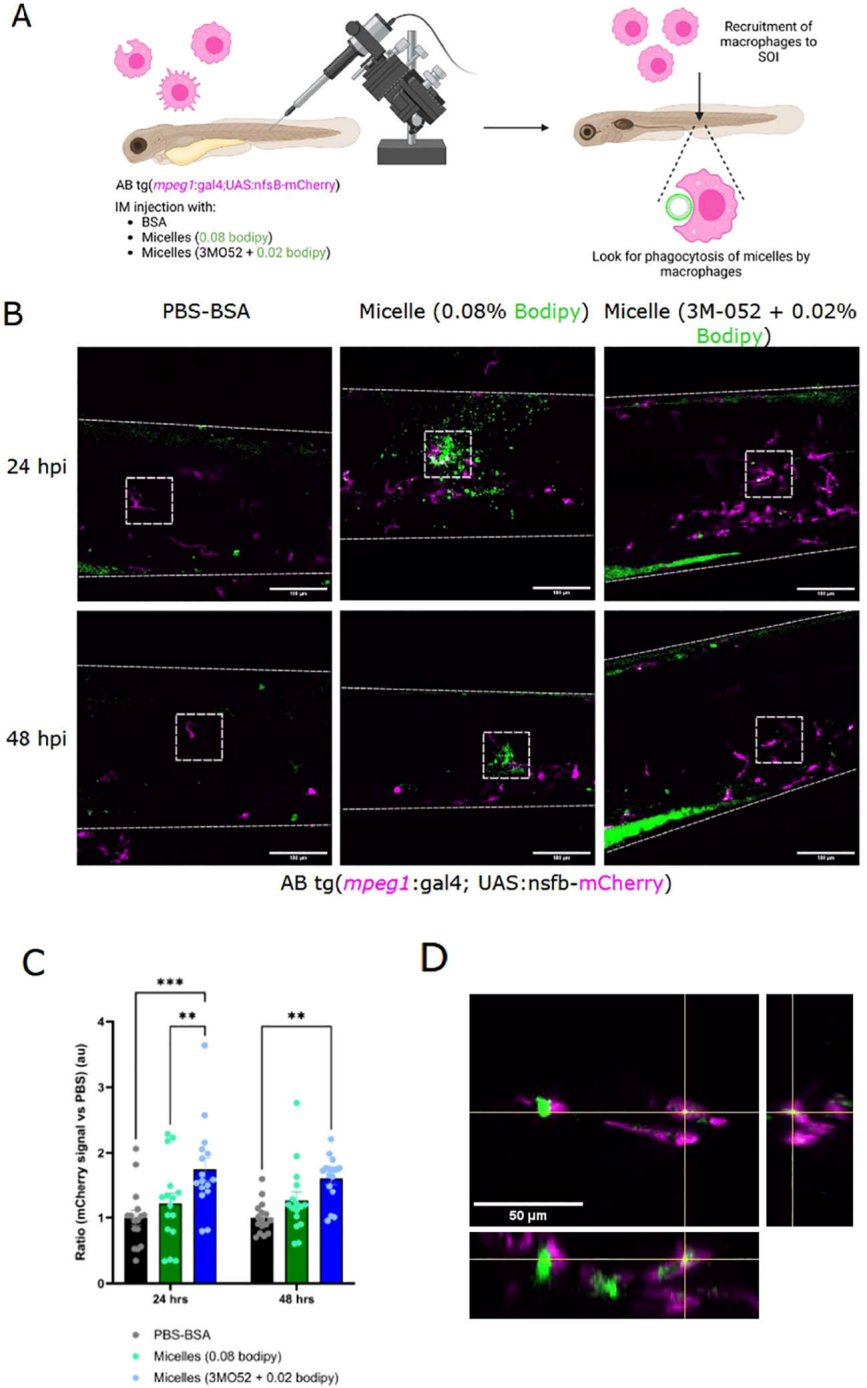
Macrophage recruitment to site of injection and phagocytosis of micelles. **(A)** Schematic diagram of experimental design. **(B)** Tg(*mpeg1*:gal4; UAS:nfsB-mCherry) larvae were intramuscularly injected by with 1 nl of PBS-BSA, micelles (0.08% Bodipy), or 3M-052 loaded micelles (0.02% bodipy). Macrophage recruitment (magenta) to the injection site was assessed at 24- and 48hpi.Representative images from 10 imaged larvae per group. **(C)** Bar plots showing the mean grey value of mCherry (macrophage) signal in arbitrary units (au) at the site of injection (White dashed box) in zebrafish at different time points. Bar plots show mean + SEM (*n* = 8 per group). Statistical significance is indicated by ** P < 0.01 and *** P < 0.001. **(D)** Orthogonal projections of a confocal Z-stack depicting a macrophage (magenta) phagocytosing micelles-loaded with 3M-052 (green) at 24 hpi.

To investigate the potential role of macrophages in the clearance of micelles at the site of injection, we used the macrophage reporter line, tg(mpeg1:gal4; UAS:nfsB-mCherry) (29) (**Figure 2A**). Macrophage recruitment was assessed by quantifying the mCherry signal at the injection site using confocal microscopy (**Figure 2B**). Expression of mCherry was significantly increased 24 and 48 hpi in fish injected with 3M-052-loaded micelles than those injected with PBS-BSA (**Figure 2C**). At 24 hpi, fish injected with micelles-loaded with 3M-052 displayed significantly higher mCherry expression compared control micelle-injected fish, demonstrating the adjuvant’s role in attracting macrophages. Furthermore, confocal imaging (**Figure 2D**) confirmed the internalization of 3M-052-containing micelles by macrophages (**Video S1**).

**Figure 3:**
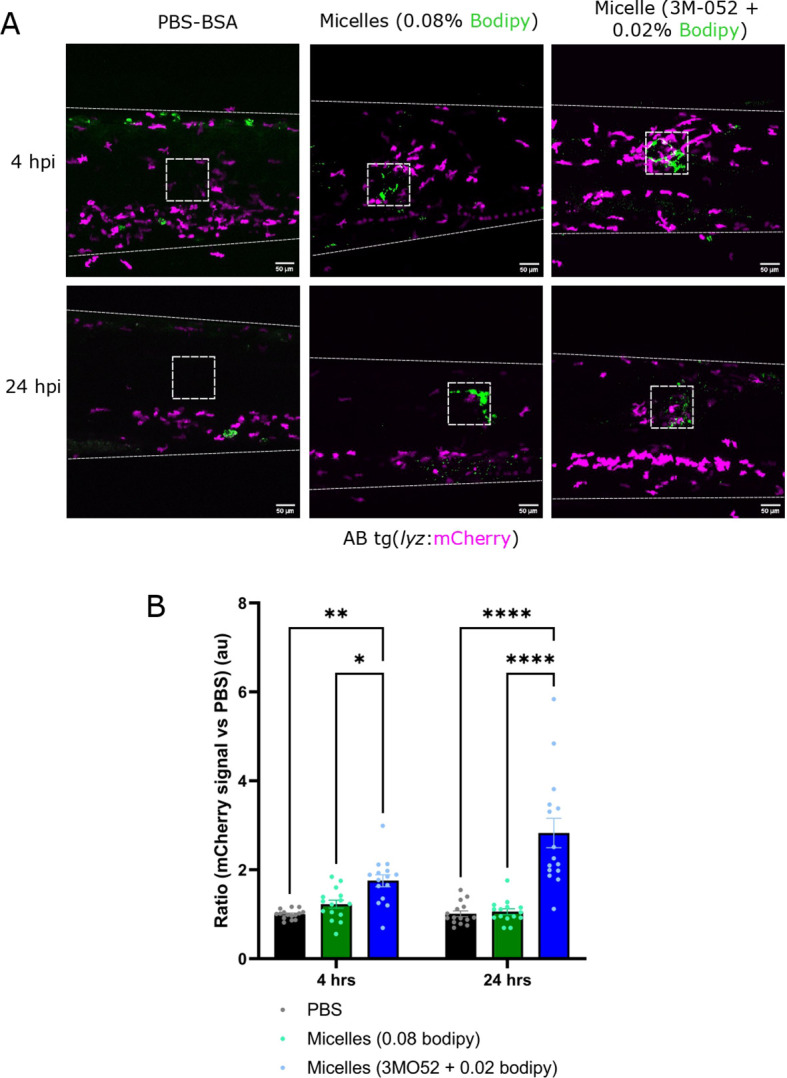
Recruitment of neutrophils to the site of injection. **(A)** Tg(*Lyz*: mCherry) larvae were intramuscularly injected by with 1 nl of PBS-BSA, micelles (0.08% Bodipy), or micelles-loaded with 3M-052 (0.08% Bodipy) (green). Neutrophil recruitment (magenta) to the injection site was assessed at 4- and 24 hpi. Representative images from 7 to 9 imaged larvae per group. **(B)** Bar plots showing the mean gray value of mCherry (neutrophil) signal in arbitrary units (au) at the site of injection (White dashed box) in zebrafish at different time points. Bar plots show mean + SEM (n = 15 per group). Statistical significance is indicated by * P < 0.05, ** P < 0.01, and **** P < 0.0001.

To assess the local neutrophil response to 3M-052-loaded micelles, the tg(*lyz*:nsfb-mCherry) (30) neutrophil reporter line was used (**Figure 3A**). Neutrophils are known to be rapidly recruited to the site of infection to clear any breaching pathogens, as such to capture early recruitment and persistence of neutrophils to the site of injection imaging time points were focused on shorter term time points compared to macrophages at 4 and 24 hpi. Larvae were injected IM as above. At 4 hpi, mCherry fluorescence was significantly increased in the 3M-052 group compared to BSA controls. This elevated signal was sustained at 24 hpi, with significantly higher mCherry fluorescence, still being observed at the injection site, in the fish injected with micelles loaded with 3M-052 compared to both the empty micelles and BSA controls. No difference was seen between the BSA and empty micelle groups at either time point (**Figure 3B**).

Despite robust recruitment, we did not observe positive evidence of micelle internalization by neutrophils. Neutrophils were frequently detected in close proximity to the 3M-052 group; however, fluorescence microscopy did not reveal definitive co-localization consistent with intracellular uptake. These findings suggest that, in this context, neutrophils are primarily recruited to the site of injection as a result of 3M-052 and may contribute to local inflammatory signaling rather than acting as major phagocytic cells for micelle uptake.

Together these data indicate that 3M-052 delivered via micelles drives a sustained macrophage and neutrophil chemoattractive response at the site of injection, consistent with the idea that 3M-052 drives localized innate immune activity.

### Limited ISG gene expression response to micelles and 3M-052-containing micelles in AB and myd88 mutant zebrafish

**Figure 4:**
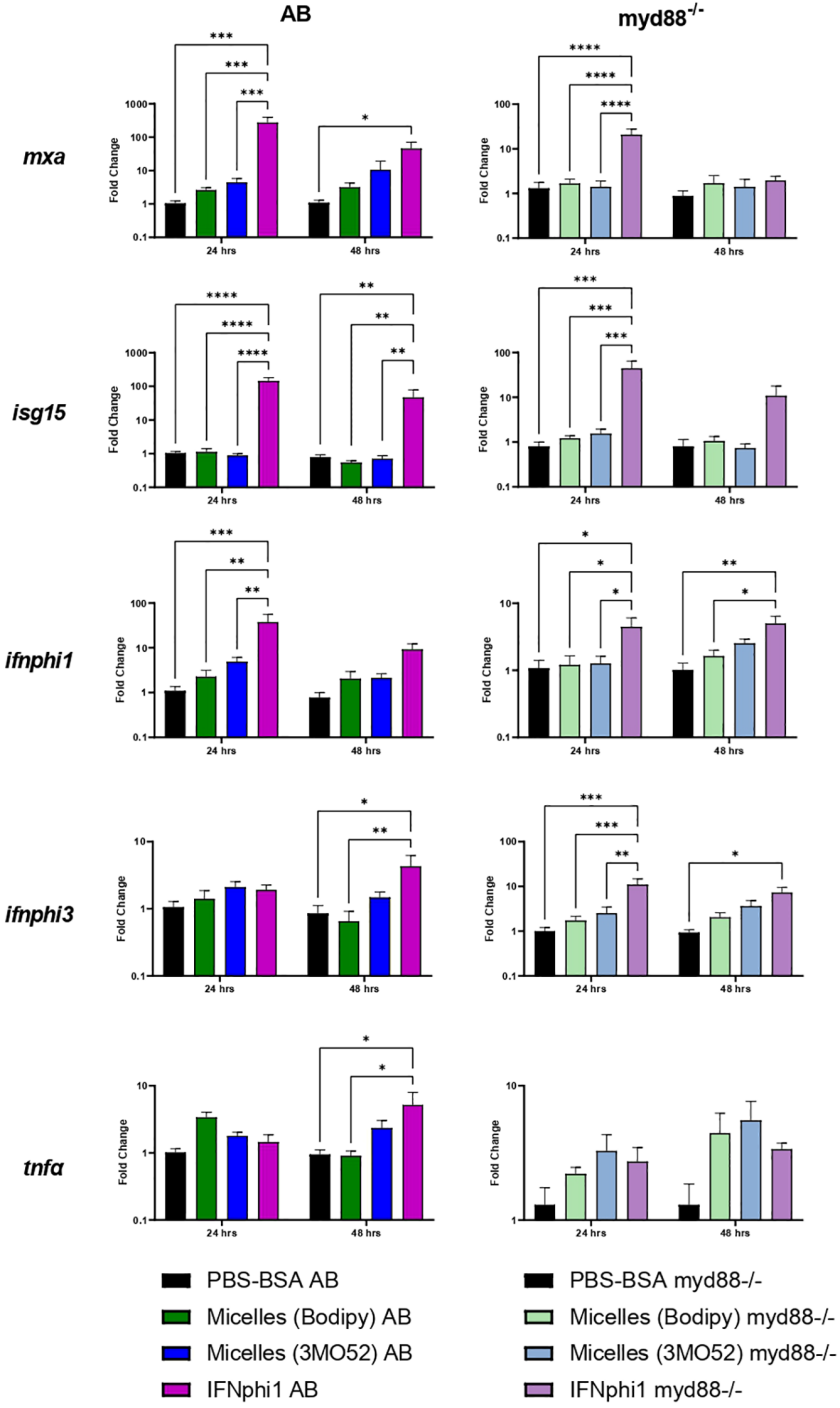
Whole-body qRT-PCR analysis of response to micelles do not reveal a systemic response. qPCR analysis of *mxa, isg15, ifnphi1* (secreted form)*, ifnphi3*, and *tnfa* gene expression in AB or myd88 mutant zebrafish at 24 and 48 hpi with different formulations. The y-axis represents fold change in gene expression (log scale) relative to PBS-BSA controls. Data are presented as mean with SEM. For each sample 3 zebrafish were pooled before RNA extraction and *n* = 9 per group. Statistical significance is indicated by * P < 0.05, ** P < 0.01, *** P < 0.001, and **** P < 0.0001.

To evaluate the immune response to micelles and 3M-052-loaded micelles, we quantified the expression of five immune-related genes; two IFN-stimulated genes (ISGs), *mxa*, and *isg15*; two type I IFNs, *ifnphi1* (secreted isoform) *and ifnphi3*, and the pro-inflammatory cytokine *tnfa*, in whole zebrafish larvae by qPCR at 24- and 48-hpi (**Figure 4**). Since 3M-052 triggers TLR7/8 responses which require the Myd88 adaptor, we compared wild-type (AB) and *myd88*^-/-^ larvae (31). Recombinant zebrafish IFNφ1 (rIFNφ1) was also injected as a positive control. In AB fish, while rIFNφ1 induced a strong ISG response as expected, micelles loaded with 3M-052 did not induce any significant change in expression of any gene tested, indicating they do not induce a strong systemic immune response, be it in wild-type or in *myd88*^-/-^ fish. Interestingly, some differences in the response to rIFNφ1 were observed in *myd88*-deficient larvae, such as a more rapid induction of *ifnphi3*, suggesting the existence of a Myd88 dependent negative feedback loop in the response downstream type I IFN. Overall, this analysis shows that IM injection of 3M-052 loaded micelles does not induce an appreciable systemic response.

### 3M-052-loaded micelles drive a local ISG response

**Figure 5:**
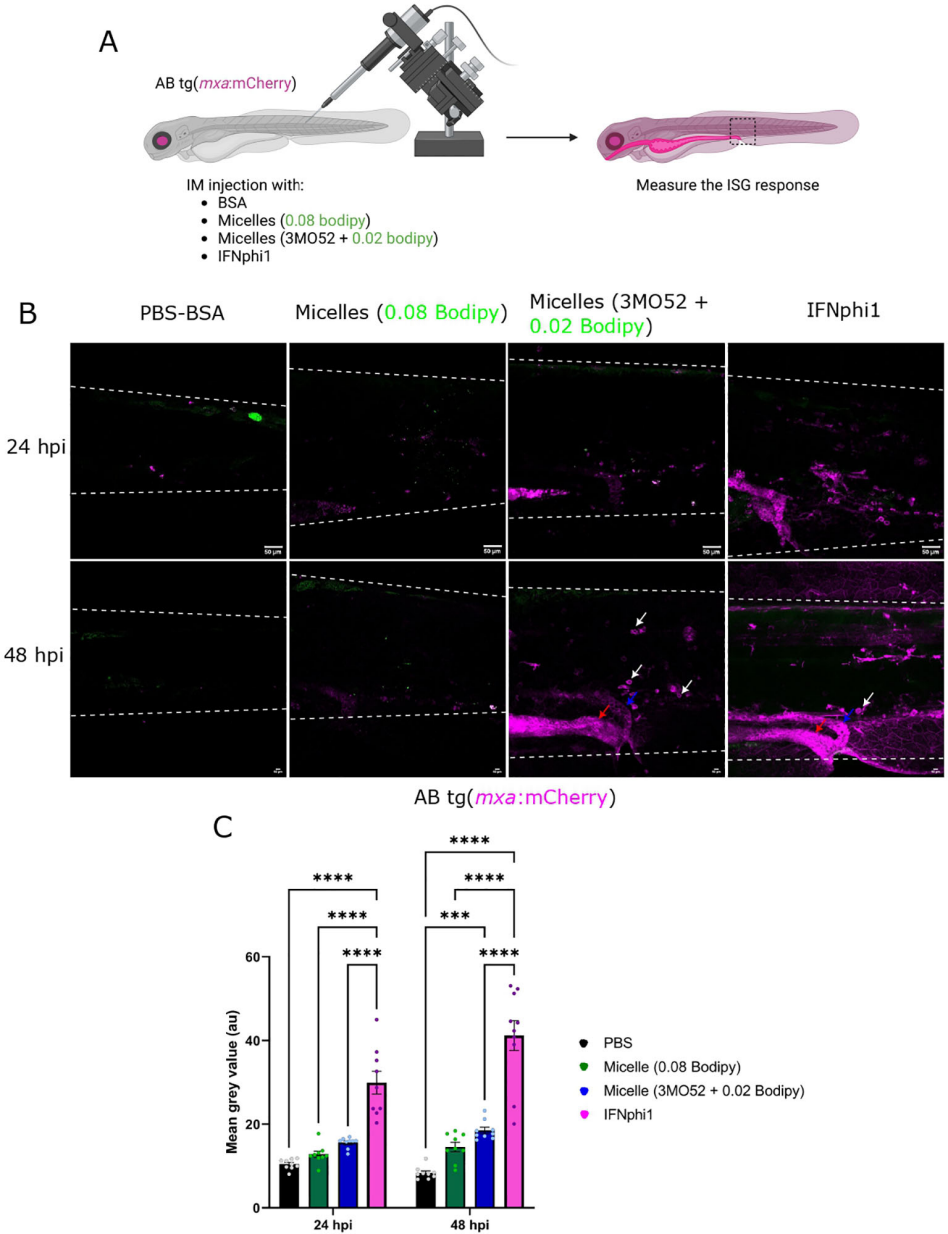
Local increase in mxa expression after injection of 3M-052-loaded micelles. **(A)** Schematic overview of experiment. **(B)** Confocal imaging showing mxa (magenta) expression after injection with PBS-BSA, micelles (0.08 Bodipy), Micelles (3M-052 + 0.02 Bodipy) or recombinant IFNφ1 at different time points. Probable macrophages (according to morphology) = white arrows, Distal gut = red arrow and distal pronephric tubule = blue arrow. Representative images from 6 (PBS, IFN), 8 (micelles), or 13 (micelles + 3M-052) imaged larvae per group. **(C)** Bar plots showing the mean gray value of mxa induction at the site of injection in zebrafish larvae. Bar plot show mean ration + SEM where *n* = 10. Statistical significance is indicated by *** P < 0.001, and **** P < 0.0001.

To assess the local immunostimulatory effects of 3M-052, a characterized ISG reporter line, tg(mxa:mCherry), was used (32) (**Figure 5A**). The tg(*mxa*:mCherry) reporter line was used due to its ability to respond to type I interferon expected to be induced downstream of the TLR7/8 signaling pathway making it a useful tool to complement gene expression data. 3M-052 loaded micelles induced a significant increase in *mxa* promoter-driven fluorescence at 2 days post-injection at the site of injection, indicating a robust but transient activation of the interferon response compared to PBS controls (**Figure 5B**). Again, rIFNφ1 served as a positive control, strongly inducing *mxa* reporter expression at both time points. In contrast, control micelles did not significantly differ from PBS injections at either 24- or 48-hpi. Levels of *mxa* induction were consistently lower, although not significantly, in zebrafish injected with micelles alone than those zebrafish injected with micelles containing 3M-052 indicating that 3M-052 is the primary driver of immune activation rather than the micelle formulation itself (**Figure 5C**). The morphology of mxa-expressing cells near to the injection site of 3M-052-loaded micelles suggest they are macrophages (**Figure 5B**; white arrows). Transgene expression is also observed in the epithelium of the distal gut (**Figure 5B**; red arrow) and distal pronephric tubule (**Figure 5B**; blue arrow), which are only 200µm distant from the injection point and are the tissues that respond most strongly to rIFNφ1 (33).

### Micelles with 3M-052 limit Sindbis virus replication at the site of injection

**Figure 6:**
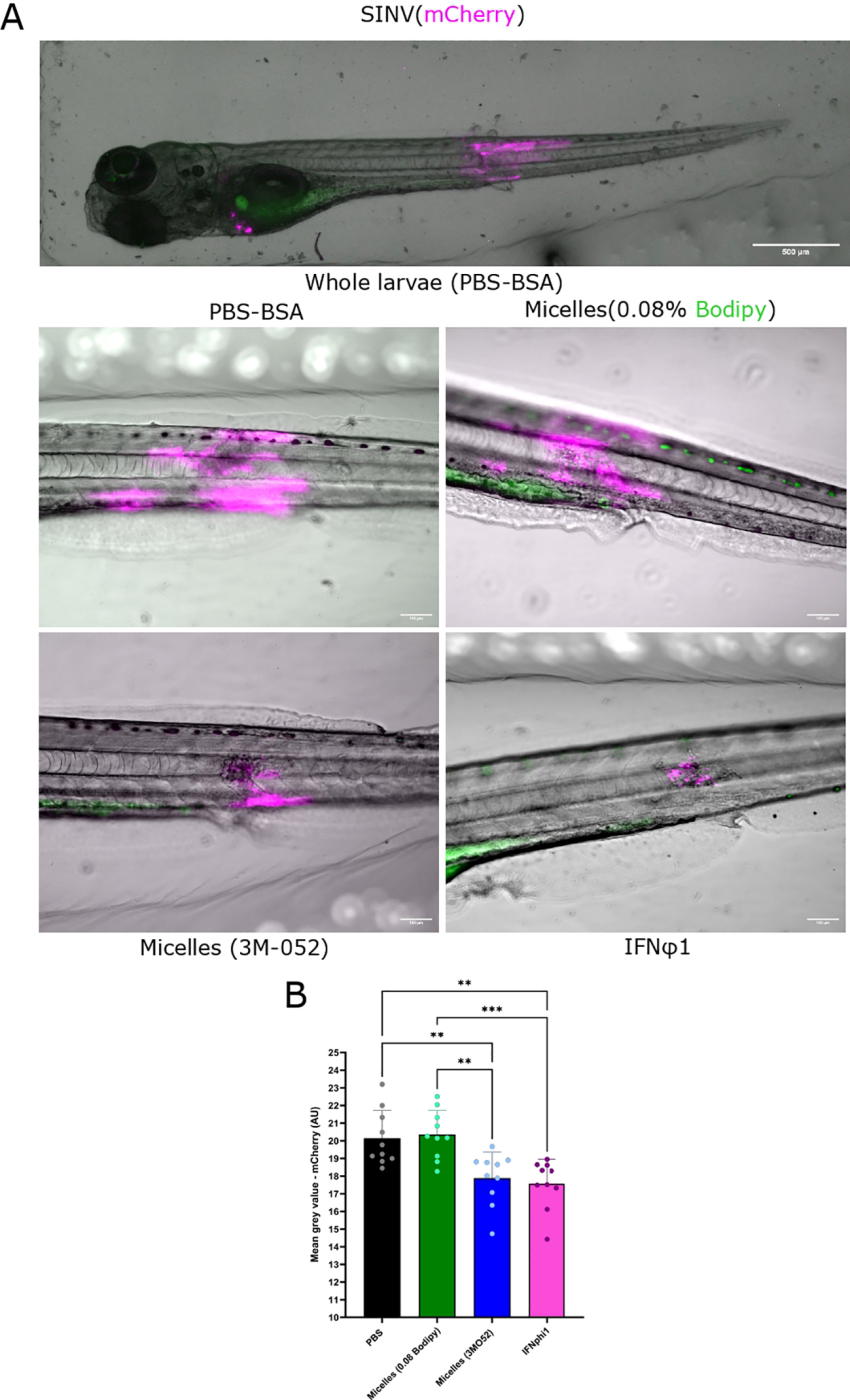
Effects of 3M-052 administration on Sindbis virus replication. **(A)** Live widefield images showing SINV replication (magenta) at 1-day post-infection (dpi). Larvae were pre-injected 1 day prior with micelles, micelles-loaded with 3M-052, IFNφ1, or PBS. Representative images from 10 imaged larvae per group. **(B)** Bar plots quantifying SINV replication by measuring the mean gray value (au) of SINV across the whole larva and at the infection site at 24 hpi. Bar plots show mean + SEM where *n* = 10 per group. Statistical significance is indicated by ** P < 0.01 and *** P < 0.001.

To assess the effect of micelles-loaded with 3M-052 on viral replication, zebrafish were first injected with micelles loaded with or without 3M-052, or with rIFNφ1 as a positive control, and challenged 24h later on the contralateral side with Sindbis virus (SINV) encoding mCherry (magenta) (SINV-mCherry). mCherry fluorescence was used to quantify viral replication across groups (**Figure 6A**), SINV was chosen because we have previously established that type I IFNs play a key role in controlling SINV replication in zebrafish (34, 35). Quantification of the infected area around the site of injection revealed that zebrafish injected with either IFNφ1 or micelles loaded with 3M-052 showed a significant decrease in viral replication (**Figure 6B**). This data supports the notion that localized type I IFN response close to the site of injection induced by 3M-052 has a significant antiviral effect.

## Discussion

In this study, we assessed the induction and efficacy of the innate antiviral immune response triggered by micelles loaded with the TLR7/8 agonist 3M-052 in zebrafish. Our results demonstrate that while micelles alone induce minimal immune activation, 3M-052-loaded micelles promote a localized interferon responses and myeloid cell recruitment at the injection site. Furthermore, 3M-052-loaded micelles effectively limited SINV replication at the injection site, indicating a functional antiviral effect similar to that of rIFNφ1 injection. However, we observed no significant systemic upregulation of ISGs, indicating that the response remains largely localized as seen in mammalian models (19).

Our findings demonstrate the intramuscular delivery of 3M-052 micelles in zebrafish results in a restricted biodistribution, primarily around the site of injection where some became internalized by macrophages. The tracking of fluorescent micelles in live zebrafish over time confirmed that the micelles remain largely confined to the site of injection over time, with some dispersal presumably due to macrophages migrating into neighboring tissues after micelle internalization. This spatial restriction with bodipy signal decreasing over time aligns with the observed immune cell dynamics where neutrophils were rapidly recruited after 4hrs and remained elevated after 24 hrs., meanwhile macrophages were also consistently present at both 24 and 48 hrs. post injection compared to PBS-BSA controls. The presence of innate immune cells is consistent a focused, local inflammatory response without systemic activation.

Micelle formulation likely contributes to this effect by promoting local retention and shielding the adjuvant from degradation, enabling a prolonged released and subsequently sustained immune stimulation at the site of injection. The slow release mechanism of micelles has been demonstrated in several studies, lasting up to two weeks (36, 37). In our model, the persistence of Bodipy signal up to 48 hours after injection supports the notion of a slow release mechanism, efficient at enhancing local immune activation while minimizing off-target or systemic exposure. Delivery systems such as these could be useful in mucosal contexts where barrier integrity and enzymatic degradation pose challenges to conventional adjuvants and vaccine delivery systems (38, 39). Future studies comparing different micelle structures and a range of adjuvant formulations will be essential to determine if the slow release effect is driven by micelle composition, specific adjuvant properties or a combination of both.

The induction of interferon at the site of injection by 3M-052-loaded micelles was most evident when zebrafish were challenged with Sindbis virus. A localized reduction in SINV-mCherry replication was induced at the injection site by 3M-052-loaded micelles, similar to that caused by rIFNφ1. This suggests that while 3M-052 delivery may not trigger systemic interferon responses, it is capable of generating an effective localized antiviral response, reinforcing the potential of micelle formulated TLR agonists in site specific immunity. Despite the local immune activation, we detected no significant systemic cytokine induction in either AB or *myd88* mutant zebrafish. This aligns with previous work demonstrating that 3M-052 does not induce a systemic response (19). Several papers have demonstrated that both TLR7 and TLR8 can be expressed in the larval stages of zebrafish development, as early as 12 hpf (40, 41), as such it is conceivable that immune cells are detecting 3M-052 through TLR7/8 detection similar to mammalian systems. In our mxa reporter line data, cellular morphology indicates that macrophages are activated by the 3M-052 loaded micelles, consistent with the evidence showing phagocytosis of micelles by macrophages.

Future research should investigate alternative TLR7/8 agonists as they have also been shown to act as strong targets for adjuvants. In zebrafish, other imidazoquinolines, such as resiquimod (R848), have been shown to drive innate immune responses in lymphocyte-like cell populations and macrophages in *rag ^-/-^* mutant zebrafish (42) whilst also offering some protection against bacterial pathogens potentially through NK cell activation (43). In mammals, other imidazoquinolines, such as gardiquimod, have been shown to improve immune cell activation, enhance lymph node targeting and induce broader immune responses when delivered in a nanoparticle formation in mice (44). When combined with influenza or SARS-CoV-2 vaccines, gardiquimod-based formulations maintained strong antibody responses. Incorporation of imidazoquinolines into the ionizable lipid of lipid nanoparticles (LNPs) of a SARS-COV2 vaccine has also demonstrated the ability of these compounds to induce strong innate responses through Th1 mediated pathways leading to strong B cell and antibody responses. Alternatively, other delivery systems, such as polymer-nanoparticle (PNP) hydrogels, may also be an interesting avenue to explore. For example 3M-052 adjuvanted PNP hydrogels demonstrating increased the magnitude, breadth and duration of humoral immune responses, whilst also improving the systemic T cell response in mice (45).

While our study primarily focused on the innate immune response to the micelle-based delivery of 3M-052, the downstream effects on the adaptive immune system remain an important area for future research. The recruitment of macrophages to the injection site suggests potential for antigen presenting cell activation, which could facilitate the priming of the adaptive immune response. In mammals, TLR7/8 agonists are known to enhance dendritic cell maturation and promote Th1-skewed adaptive immunity, characterized by cytotoxic T cell responses and a shift towards IgG2A production (46–51). Given that zebrafish and other teleost fish possess a functional adaptive immune system, it is conceivable that 3M-052-loaded micelles may similarly enhance antigen-specific responses, particularly through increased cross-presentation of viral or vaccine antigens (47). Thus, targeted IFN induction via TLR7/8 agonists like 3M-052 may not only trigger strong local innate responses but also provide a mechanistic bridge toward improved vaccine-driven adaptive immunity in aquatic species. Future studies should address antigen specific T and B cell activation in older fish following vaccinations with micelle formulations containing different adjuvants to understand whether these mechanisms remain the same as mammals.

## Limitations of the study

This study was conducted in zebrafish larvae, which lack a fully developed adaptive immune system at this stage. As a result, the interplay between recruited antigen-presenting cells (APCs) and systemic immune responses may differ from that observed in adult fish. Additionally, this work did not model a vaccine context, as no antigen was co-injected to directly assess adjuvant effects. A comparison to non-encapsulated 3M-052 was also not included, as the primary aim was to evaluate the feasibility and performance of micelle-based delivery systems in the zebrafish larvae model.

## Materials and methods

### Ethical statement

Animal experiments described in the present study were conducted according to European Union guidelines for handling of laboratory animals and were approved by the Ethics Committee of Institut des Neurosciences Paris-Saclay.

### Zebrafish lines

All zebrafish lines were raised in the aquatic facilities of the Institut des Neurosciences Paris-Saclay. WT AB zebrafish were initially obtained from ZIRC (Eugene, OR, USA). The following transgenic and mutant lines were also used: tg(*mxa*:mCherry) (32), tg(*mpeg1*:gal4; UAS:nfsB-mCherry) (29), tg(*lyz*:nsfb-mcherry) (30)and a *myd88^-/^*mutant (31). The myd88-/- were generated after 5 back-crosses onto the AB background. After natural spawning, eggs were collected, and bleached for 5 minutes using 0.03% bleach, rinsed twice using embryo medium (EM) and incubated in EM containing 0.3 μg methylene blue at 28°C. After 24 hours the EM was replaced with EM containing 200 μM phenylthiourea (PTU, Sigma) to reduce pigmentation of larvae. Zebrafish were incubated at 24°C or 28°C depending on the required developmental speed.

Xanthophore-disrupted mutants were generated by injecting fertilized eggs at the 1-cell stage with 1 nL of slc2a11b guide RNAs (5′–GTCATTGGACGCTTCCTGAC–3′, PAM: AGG [guide AB]; 5′–GAGGAGGGTTAACATATCAC–3′, PAM: TGG [guide AD]). Although slc2a11b encodes a transporter expressed in xanthophores, its disruption does not eliminate the xanthophore population. Eggs were otherwise treated as described above.

### Micelles

Micelles from polylactide-b-poly(N-acryloxysuccinimide-co-N-vinylpyrrolidone) copolymer (mean size: 80 ± 4 nm, polydispersity index: 0.040 ± 0.003, by dynamic light scattering) were prepared as previously reported (52). In brief, 20 mg of copolymer were dissolved in 2 mL acetonitrile (Carlo Erba Reagents) and 8 µL of Bodipy 500/510 C4 C9 (ThermoFisher Scientific, 2 mg.mL-1 in ethanol), namely 0.08 wt% compared to copolymer, were added to the solution. This organic solution was then added to 4 ml of pure water under agitation (200 rpm), allowing the formation of micelles. Acetonitrile and a part of water were removed by evaporation under reduced pressure. The micelle concentration of the prepared micelles was determined by measuring the solid content, after heating a known and sacrificed volume of the micellar solution to constant weight in an oven at 70 °C for 24 h. Based on this, micelle concentration was adjusted to 5 mg.mL-1 by adding dedicated volume of water. Bodipy encapsulation was directly evidenced on the micellar solution by visible spectrometry using a Tecan i-control Infinite M1000 (Tecan, Männedorf, Switzerland), and encapsulation efficiency (~100%) was determined by visible spectrometry after 10-fold dilution in acetonitrile, using a calibration curve established under the same conditions, as previously described (53) Telratolimod (3M-052, MedChemExpress) was loaded into the micelles and quantified by UV-visible spectrometry as previously described in (52) for imiquimod, with an effective drug loading of 6 µg/mg of micelle.

### Viruses

The SINV-mCherry virus, as previously described in (35), was used as a 6.10e (8) PFU/mL suspension, which was diluted with an equal volume of 0.5% phenol red solution (Sigma) to facilitate inoculum visualization 1 nL of inoculum containing about 30 PFUs. A single independent virus challenge experiment was performed where n=10 per group.

### Injections

Zebrafish larvae aged 3- or 4-days post-fertilization (dpf), depending on the experiment, were microinjected with approximately 1 nL of either micelle (5 mg/mL^-1^, loaded or not with Bodipy and/or 3M-052), recombinant IFNφ1 (1 ng/μL in PBS), BSA (1 ng/μL in PBS), or Sindbis virus (30 pfu). Prior to injection, larvae were anesthetized with 0.2 mg/mL tricaine and positioned on 1% low-melting agarose plates with pre-molded grooves for consistent orientation. Microinjections were performed intramuscularly into the somatic muscle using a micromanipulator for all injections. Following injection, larvae were transferred to culture plates containing embryo medium supplemented with PTU and maintained at 28 °C until imaging. All zebrafish larvae were euthanized at 5 dpf by tricaine overdose.

### Lysis, RNA extraction and RTqPCR of larvae

RNA was extracted from pooled larvae with 3 larvae per extraction. Larvae were culled immediately before lysis by overdose of tricaine. The EM was then removed and replaced with 300 μL of RLT buffer (Qiagen) before mechanical dissociation by pipetting. Lysates were then stored in tubes at -20°C until RNA extraction could take place. Total RNA was extracted using the RNA easy mini kit (Qiagen) following the manufacturer’s instructions and was eluted in 20 μL of nuclease free water.

Reverse transcription was performed on 10 μL of eluted RNA using the SuperScript IV Reverse Transcriptase kit with dT_17_ primer (for polyadenylated transcripts). cDNA was diluted to a final volume of 200 μL (10x), of which 5 μL was used as a template for each qPCR assay.

Real-time qPCR was performed using the QuantStudio™ 5 System with the following cycling: 50 °C for 2 mins, 95 °C for 10 mins, 40 cycles of 95 °C for 15s and 60 °C for 1 minute followed by a melt curve analysis. Quantification was carried out using a SYBR assay using the PowerUp SYBR Green master mix (ThermoFisher Scientific) with primer pairs (**Table 1**) (54–57). Normalization of the results was performed using the housekeeping gene *elongation factor 1-alpha 1* (*eef1a1l1*).

### Live widefield fluorescence imaging

Live widefield fluorescence imaging was performed using an EVOS FL microscope (Thermo Fisher) with either a 2× objective for whole-larvae imaging or a 10× objective for detailed views of the injection site. Before imaging, larvae were anesthetized in 0.2 mg/mL tricaine and positioned laterally in individual wells of a 1% agarose mold. Transmitted light and fluorescence images (GFP or Texas Red) were acquired. After imaging, larvae were rinsed in EM, transferred back to Petri dishes containing EM, and incubated at 28 °C until further imaging.

### Live confocal imaging

Confocal imaging was performed using a Leica SP8 confocal microscope. Larvae were anesthetized in 0.2 mg/mL tricaine, mounted laterally in 1% agarose molds, and immobilized with 1% low-melting-point agarose before being submerged in EM containing 0.02 mg/mL tricaine. Imaging was conducted using a 20× water objective, with mCherry and BODIPY 510 excited by 561 nm and 488 nm lasers, respectively. Z-stacks of the injection site were acquired. After imaging, larvae were removed from their mounts, rinsed, and stored in EM at 28 °C for further imaging.

### Image analysis

Image analysis was performed using the Fiji software (58). EVOS images were normalized and thresholded, ensuring consistent channel settings across all experiments. Confocal Z-stacks were processed using Fiji, including normalization and maximal projections. Fluorescence intensity (mean grey value) was quantified from normalized images or maximum projections to ensure consistent and accurate measurements in Fiji. For analysis at the injection site, a region of interest (ROI) was defined based on the initial micelle spread, and a fixed area (~100 × 100 µm) was subsequently applied across all conditions, time points, and reporter lines to enable direct comparison of fluorescence intensity.

### Statistical analysis

Statistical analysis was carried out in Graphpad prism 9. A two-way ANOVA with a Tukey’s *post hoc* test was used to confer statistical differences between groups when comparing fluorescence intensity.

## Data Availability

The datasets presented in this study can be found o the Zenodo online repository, under DOI: 10.5281/zenodo.20539476.

## Supplementary material

Supplementary Figure 1 Effect of slc2a11b Mutation on Pigmentation **(A)** Comparison of pigmentation between wild-type (AB) and *slc2a11b* mutant zebrafish larvae at 5 days post-fertilization (dpf). **(B)** Pigmentation differences observed 1-day post-injection (dpi; corresponding to 4 dpf) with micelles (0.08% Bodipy).

Supplementary Video 1 3D Projection of micelle uptake by Macrophages. projection showing macrophages (magenta) engulfing micelles containing 3M-052 (green) at 1 day post-injection (1 dpi) in *Tg(mpeg1:Gal4; UAS:nfsB-mCherry)* zebrafish larvae.

Supplementary Table 1 qPCR primers used in this study.
