# Supplementary material for: TLR7/8 agonist 3M-052 formulated in micelles induces local type I IFN response and antiviral immunity in zebrafish larvae
Source: Front Immunol. 2026 Jun 16;17:1834678. doi: 10.3389/fimmu.2026.1834678 (PMC13314456; doi:10.3389/fimmu.2026.1834678)
Supplement: Supplementary Figure 1 — Effect of slc2a11b Mutation on Pigmentation (A) Comparison of pigmentation between wild-type (AB) and slc2a11b mutant zebrafish larvae at 5 days post-fertilization (dpf). (B) Pigmentation differences observed 1-day post-injection (dpi; corresponding to 4 dpf) with micelles (0.08% Bodipy). [file DataSheet1.pdf]

Supplementary information to Porter et al.

Localized innate antiviral immunity induced in zebrafish by the synthetic adjuvant 3M-052 embedded in micelles

Figure S1 – Effect of slc2a11b Mutation on Pigmentation A Comparison of pigmentation between wild-type (AB) and *slc2a11b* mutant zebrafish larvae at 5 days post-fertilization (dpf). **B** Pigmentation differences observed 1-day post-injection (dpi; corresponding to 4 dpf) with micelles (0.08% Bodipy).

Video S1 – 3D Projection of micelle uptake by Macrophages. projection showing macrophages (magenta) engulfing micelles containing 3M-052 (green) at 1 day post-injection (1 dpi) in *Tg(mpeg1:Gal4; UAS:nfsB-mCherry)* zebrafish larvae.

Supplementary Table 1 – qPCR primers used in this study

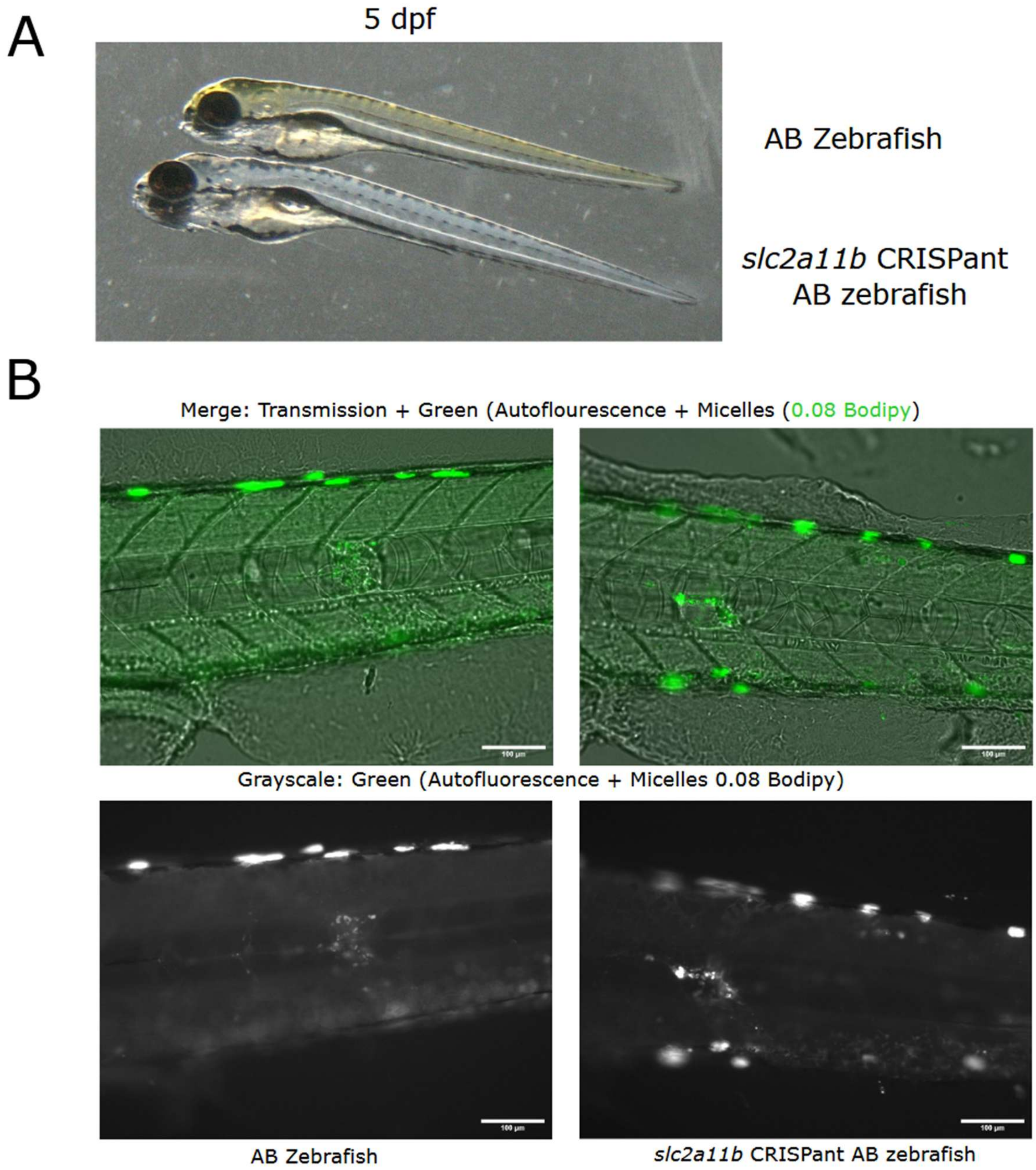
A
5 dpf
AB Zebrafish
slc2a11b CRISPant
AB zebrafish
B
Merge: Transmission + Green (Autoflourescence + Micelles (0.08 Bodipy)
Grayscale: Green (Autofluorescence + Micelles 0.08 Bodipy)
AB Zebrafish
slc2a11b CRISPant AB zebrafish

Figure S1

Supplementary Table 1 – qPCR primers used in this study

Gene Name	Gene ID	Forward Primer (5' - 3')	Reverse Primer (5' - 3')	Reference
Elongation factor 1 alpha 1	*eef1a1l1*	GCTGATCGTTGGAGTCAACA	ACAGACTTGACCTCAGTGGT	[55]
myxovirus (influenza) resistance A	*mxa*	GACCGTCTCTGATGTGGTTA	GCATGCTTTAGACTCTGGCT	[54]
ISG15 ubiquitin like modifier	*isg15*	AACTCGGTGACGATGCAGC	TGGGCACGTTGAAGTACTGA	[56]
interferon phi 3	*ifnphi3*	GAGGATCAGGTTACTGGTGT	GTTCATGATGCATGTGCTGTA	[54]
Interferon phi 1	*ifnphi1*	TGAGAACTCAAATGTGGACCT	GTCCTCCACCTTTGACTTGT	[54]
Tumour Necrosis Factor alpha	*tnfα*	TTCACGCTCCATAAGACCCA	CAGAGTTGTATCCACCTGTTA	[57]
